# 2-Bromo-4-chloro-6-(4-fluoro­phenyl­imino­meth­yl)phenol

**DOI:** 10.1107/S1600536808017443

**Published:** 2008-06-25

**Authors:** G. Puthilibai, S. Vasudhevan, G. Rajagopal

**Affiliations:** a Department of Chemistry, Bharath University, Chennai 600 073, India; b Department of Chemistry, KV Central Leather Research Institute, Chennai 600 020, India; c Department of Chemistry, Government Arts College (Men), Nandanam, Chennai 600 035, India

## Abstract

The two mol­ecules of the title compound, C_13_H_8_BrClFNO, in the asymmetric unit are inter­connected by π–π inter­actions between the salicylaldehyde and aniline units, the shortest inter­planar distance being 3.317 (3) Å. These pairs and their translation equivalents are further linked by C—H⋯F hydrogen bonds, forming a one-dimensional infinite chain.  In addition, there is an intra­molecular O—H⋯N hydrogen bond connecting the OH group and the imine N atom.

## Related literature

For related literature, see: Collinson & Fenton (1996 ▶); Garnovski & Vasil Chenko (2002 ▶); Kannan & Ramesh (2006 ▶); Karvembu *et al.* (2003 ▶); Kumar & Ramesh (2004 ▶); Nakajima *et al.* (1998 ▶); Prabhakaran *et al.* (2004 ▶); Ramesh & Maheswaran (2003 ▶); Sivagamasundari & Ramesh (2007 ▶).
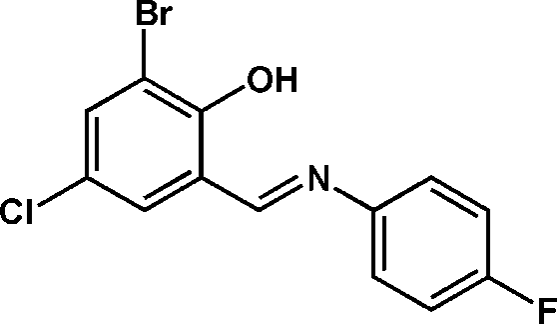


## Experimental

### 

#### Crystal data

C_13_H_8_BrClFNO
*M* *_r_* = 328.56
Triclinic, 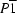

*a* = 8.2274 (3) Å
*b* = 8.6566 (3) Å
*c* = 10.8880 (4) Å
α = 69.545 (2)°
β = 70.820 (2)°
γ = 62.341 (2)°
*V* = 630.48 (4) Å^3^
*Z* = 2
Mo *K*α radiation
μ = 3.47 mm^−1^
*T* = 293 (2) K
0.30 × 0.20 × 0.20 mm

#### Data collection

Bruker APEX2 CCD diffractometer
Absorption correction: multi-scan (*SADABS*; Bruker, 1999 ▶) *T*_min_ = 0.451, *T*_max_ = 0.573 (expected range = 0.393–0.500)
16111 measured reflections
3975 independent reflections
2533 reflections with *I* > 2σ(*I*)
*R*_int_ = 0.027

#### Refinement

*R*[*F* ^2^ > 2σ(*F* ^2^)] = 0.044
*wR*(*F* ^2^) = 0.138
*S* = 0.99
3975 reflections
164 parameters
H-atom parameters constrained
Δρ_max_ = 0.72 e Å^−3^
Δρ_min_ = −0.56 e Å^−3^

### 

Data collection: *APEX2* (Bruker, 2004 ▶); cell refinement: *APEX2* and *SAINT* (Bruker, 2004 ▶); data reduction: *SAINT* and *XPREP* (Bruker, 2004 ▶); program(s) used to solve structure: *SIR92* (Altomare *et al*., 1993 ▶); program(s) used to refine structure: *SHELXL97* (Sheldrick, 2008 ▶); molecular graphics: *ORTEP-3* (Farrugia, 1997 ▶); software used to prepare material for publication: *SHELXL97*.

## Supplementary Material

Crystal structure: contains datablocks global, I. DOI: 10.1107/S1600536808017443/im2067sup1.cif

Structure factors: contains datablocks I. DOI: 10.1107/S1600536808017443/im2067Isup2.hkl

Additional supplementary materials:  crystallographic information; 3D view; checkCIF report

## Figures and Tables

**Table 1:** Hydrogen-bond geometry (Å, °)

*D*—H⋯*A*	*D*—H	H⋯*A*	*D*⋯*A*	*D*—H⋯*A*
C11—H11⋯F1^i^	0.93	2.45	3.349 (4)	162
O1—H1⋯N1	0.82	1.86	2.577 (3)	146

Symmetry code: (i) 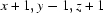.

**Fig. 1.**
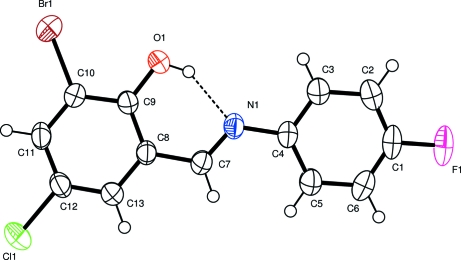
The ORTEP representation of the molecule with thermal ellipsoids at the 50% probability level.

**Fig. 2.**
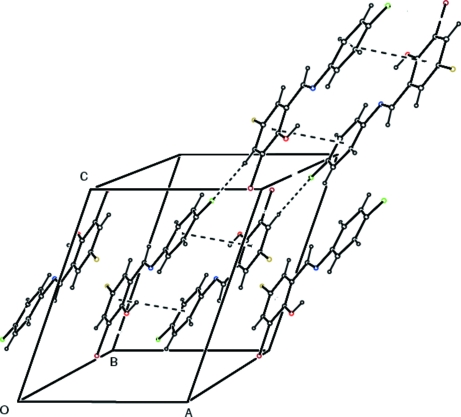
Packing of molecules in the unit cell. Intermolecular interactions are shown with dashed lines.

## supplementary crystallographic information

### Comment

Monobasic bidentate Schiff base ligands exemplified by the title compound exhibiting both N and O donor sites play an important role in the synthesis of metal complexes and represent an important class of chelating ligands (Sivagamasundari *et al.*, 2007; Prabhakaran *et al.*, 2004). Among the prodigious number and variety of Schiff bases, salicylaldimines have been studied widely because of their synthetic proclivity and structural diversity (Collinson *et al.*, 1996; Garnovski *et al.*, 2002). In recent years, there has been considerable interest in the chemistry of transition metal complexes of Schiff bases. This is due to the fact that Schiff bases offer opportunities for inducing substrate chirality, tuning metal centered electronic properties, enhancing solubility and stability of either homogeneous or heterogeneous catalysts and producing antibacterial agents (Karvembu *et al.*, 2003; Nakajima *et al.*, 1998; Kumar *et al.*, 2004; Ramesh *et al.*, 2003; Kannan *et al.*, 2006). With the above view, in our ongoing research, we have chosen the title compound as a specific and representative ligand to synthesize ruthenium complexes. The title compound and its complexes will be screened against the bacterei *E. coli*, S.aureous, P.mirabilis and P.vulgaris.

The title compound, C_9_H_8_BrClFNO, crystallizes in the triclinic space group *P*1 with one molecule in the asymmetric unit. Figure 1 shows the *ORTEP* representation of the molecule with thermal ellipsoids at the 50% probability level. The packing of the molecules in the unit cell showing the inter molecular interactions is depicted in Figure 2. The molecule and its inversion analogue are linked to each other by Π-Π interactions between the salicylaldehyde moiety and the aniline moiety with the shortest interplanar distance of 3.317 (3) Å (1 - *x*, 1 - *y*, 1 - *z*). The molecules are further connected by C11—H11···F1 hydrogen bonds (2.452 Å, 161.89°, 1 + *x*, -1 + *y*, 1 + *z*) forming an one- dimensional infinite chain. The packing is further stabilized by Van der Waals interactions. In addition, an intramolecular hydrogen bonding O1—H1···N1 (2.577 (3) Å, 145.9°) linking the OH group of the former salicyleldehyde and the imine N atom. The dihedral angle between the salicylaldehyde and aniline moieties is 8.8 (2)°.

### Experimental

The monobasic bidentate Schiff base ligand, 2-bromo-4-chloro-6-[(4' -fluorophenylimino)-methyl]-phenol, was synthesized by the condensation of 3-bromo-5-chloro-2-hydroxybenzaldehyde (0.1 mmol) with 4-fluoroaniline (0.1 mmol) in a 1:1 molar ratio in MeOH (25 cm^3^). The solution was heated under reflux for 3 h with continuous stirring and then concentrated to 5 cm^3^. On cooling the pale orange crystalline product precipitated, was filtered off, washed with ice cold EtOH and dried. The product was recrystallized from EtOH. The purity of the compound was checked by TLC.

### Refinement

All the H atoms were located from the difference Fourier map. However, the aromatic H atoms were geometrically constrained at idealized positions (C—H = 0.93 Å) and were refined using a riding model with *U*_iso_ equal to 1.2 times *U*_eq_ of the parent carbon atom. The hydroxyl hydrogen was refined isotropically with restraint: O—H = 0.820 (1) Å.

### Crystal data

**Table:**

C_13_H_8_BrClFNO	*Z* = 2
*M**_r_* = 328.56	*F*_000_ = 324
Triclinic, *P*1	*D*_x_ = 1.731 Mg m^−^^3^
Hall symbol: -P 1	Mo *K*α radiation λ = 0.71073 Å
*a* = 8.2274 (3) Å	Cell parameters from 5635 reflections
*b* = 8.6566 (3) Å	θ = 2.7–31.1º
*c* = 10.8880 (4) Å	µ = 3.47 mm^−^^1^
α = 69.545 (2)º	*T* = 293 (2) K
β = 70.820 (2)º	Rectangle, pale orange
γ = 62.341 (2)º	0.30 × 0.20 × 0.20 mm
*V* = 630.48 (4) Å^3^	

### Data collection

**Table:**

Bruker APEX2 CCD diffractometer	3975 independent reflections
Radiation source: fine-focus sealed tube	2533 reflections with *I* > 2σ(*I*)
Monochromator: graphite	*R*_int_ = 0.027
*T* = 293(2) K	θ_max_ = 30.9º
ω and φ scans	θ_min_ = 2.0º
Absorption correction: multi-scan(SADABS; Bruker, 1999)	*h* = −11→11
*T*_min_ = 0.451, *T*_max_ = 0.573	*k* = −12→12
16111 measured reflections	*l* = −15→15

### Refinement

**Table:**

Refinement on *F*^2^	Secondary atom site location: difference Fourier map
Least-squares matrix: full	Hydrogen site location: inferred from neighbouring sites
*R*[*F*^2^ > 2σ(*F*^2^)] = 0.044	H-atom parameters constrained
*wR*(*F*^2^) = 0.138	*w* = 1/[σ^2^(*F*_o_^2^) + (0.0708*P*)^2^ + 0.3467*P*] where *P* = (*F*_o_^2^ + 2*F*_c_^2^)/3
*S* = 0.99	(Δ/σ)_max_ = 0.001
3975 reflections	Δρ_max_ = 0.72 e Å^−^^3^
164 parameters	Δρ_min_ = −0.56 e Å^−^^3^
Primary atom site location: structure-invariant direct methods	Extinction correction: none

### Special details

**Table:**

Geometry. All e.s.d.'s (except the e.s.d. in the dihedral angle between two l.s. planes) are estimated using the full covariance matrix. The cell e.s.d.'s are taken into account individually in the estimation of e.s.d.'s in distances, angles and torsion angles; correlations between e.s.d.'s in cell parameters are only used when they are defined by crystal symmetry. An approximate (isotropic) treatment of cell e.s.d.'s is used for estimating e.s.d.'s involving l.s. planes.
Refinement. Refinement of *F*^2^ against ALL reflections. The weighted *R*-factor *wR* and goodness of fit *S* are based on *F*^2^, conventional *R*-factors *R* are based on *F*, with *F* set to zero for negative *F*^2^. The threshold expression of *F*^2^ > σ(*F*^2^) is used only for calculating *R*-factors(gt) *etc*. and is not relevant to the choice of reflections for refinement. *R*-factors based on *F*^2^ are statistically about twice as large as those based on *F*, and *R*- factors based on ALL data will be even larger.

### Fractional atomic coordinates and isotropic or equivalent isotropic displacement parameters (Å^2^)

**Table:**

	*x*	*y*	*z*	*U*_iso_*/*U*_eq_	
C1	0.3576 (4)	0.9290 (4)	0.1434 (3)	0.0520 (7)	
C2	0.3513 (4)	1.0070 (4)	0.2347 (3)	0.0572 (7)	
H2	0.2789	1.1284	0.2308	0.069*	
C3	0.4551 (4)	0.9013 (4)	0.3333 (3)	0.0501 (6)	
H3	0.4523	0.9521	0.3968	0.060*	
C4	0.5627 (3)	0.7212 (3)	0.3389 (2)	0.0384 (5)	
C5	0.5679 (4)	0.6469 (4)	0.2429 (3)	0.0486 (6)	
H5	0.6412	0.5261	0.2448	0.058*	
C6	0.4637 (4)	0.7527 (4)	0.1440 (3)	0.0535 (7)	
H6	0.4662	0.7040	0.0792	0.064*	
C7	0.7801 (4)	0.4644 (3)	0.4552 (3)	0.0405 (5)	
H7	0.8117	0.4086	0.3866	0.049*	
C8	0.8715 (3)	0.3665 (3)	0.5699 (2)	0.0366 (5)	
C9	0.8321 (3)	0.4496 (3)	0.6718 (3)	0.0378 (5)	
C10	0.9237 (4)	0.3494 (3)	0.7792 (3)	0.0417 (5)	
C11	1.0468 (4)	0.1735 (3)	0.7878 (3)	0.0433 (6)	
H11	1.1053	0.1086	0.8607	0.052*	
C12	1.0820 (4)	0.0950 (3)	0.6861 (3)	0.0434 (6)	
C13	0.9977 (4)	0.1888 (3)	0.5779 (3)	0.0427 (5)	
H13	1.0248	0.1338	0.5098	0.051*	
N1	0.6581 (3)	0.6243 (3)	0.4471 (2)	0.0402 (5)	
O1	0.7120 (3)	0.6194 (2)	0.6688 (2)	0.0525 (5)	
H1	0.6570	0.6571	0.6070	0.087 (13)*	
F1	0.2529 (3)	1.0312 (3)	0.04826 (19)	0.0759 (6)	
Cl1	1.23745 (13)	−0.12851 (9)	0.69813 (9)	0.0681 (2)	
Br1	0.87756 (6)	0.45988 (5)	0.91496 (4)	0.07867 (18)	

### Atomic displacement parameters (Å^2^)

**Table:**

	*U*^11^	*U*^22^	*U*^33^	*U*^12^	*U*^13^	*U*^23^
C1	0.0463 (14)	0.0613 (17)	0.0414 (14)	−0.0219 (13)	−0.0227 (12)	0.0098 (13)
C2	0.0576 (17)	0.0432 (14)	0.0585 (18)	−0.0087 (13)	−0.0269 (14)	0.0004 (13)
C3	0.0554 (16)	0.0432 (14)	0.0507 (15)	−0.0129 (12)	−0.0241 (12)	−0.0063 (12)
C4	0.0385 (12)	0.0400 (12)	0.0367 (12)	−0.0169 (10)	−0.0153 (10)	0.0002 (10)
C5	0.0505 (15)	0.0456 (14)	0.0504 (15)	−0.0129 (12)	−0.0236 (12)	−0.0079 (12)
C6	0.0586 (17)	0.0672 (19)	0.0400 (14)	−0.0264 (15)	−0.0212 (12)	−0.0057 (13)
C7	0.0443 (13)	0.0398 (12)	0.0407 (12)	−0.0146 (10)	−0.0174 (10)	−0.0078 (10)
C8	0.0373 (11)	0.0352 (11)	0.0377 (12)	−0.0126 (9)	−0.0156 (9)	−0.0040 (9)
C9	0.0362 (11)	0.0339 (11)	0.0425 (13)	−0.0089 (9)	−0.0154 (10)	−0.0080 (10)
C10	0.0437 (13)	0.0425 (13)	0.0413 (13)	−0.0127 (10)	−0.0174 (10)	−0.0102 (10)
C11	0.0449 (13)	0.0397 (12)	0.0425 (13)	−0.0128 (10)	−0.0222 (11)	0.0005 (11)
C12	0.0429 (13)	0.0310 (11)	0.0522 (15)	−0.0083 (10)	−0.0200 (11)	−0.0044 (10)
C13	0.0471 (13)	0.0347 (12)	0.0462 (14)	−0.0100 (10)	−0.0177 (11)	−0.0102 (10)
N1	0.0409 (11)	0.0395 (11)	0.0407 (11)	−0.0139 (9)	−0.0183 (9)	−0.0034 (9)
O1	0.0568 (11)	0.0372 (9)	0.0584 (12)	0.0023 (8)	−0.0302 (9)	−0.0173 (8)
F1	0.0744 (12)	0.0842 (14)	0.0574 (11)	−0.0237 (11)	−0.0444 (10)	0.0151 (10)
Cl1	0.0768 (5)	0.0338 (3)	0.0812 (6)	0.0009 (3)	−0.0358 (4)	−0.0126 (3)
Br1	0.0981 (3)	0.0702 (3)	0.0663 (3)	−0.00208 (19)	−0.0451 (2)	−0.03146 (18)

### Geometric parameters (Å, °)

**Table:**

C1—F1	1.355 (3)	C7—H7	0.9300
C1—C6	1.361 (5)	C8—C13	1.391 (3)
C1—C2	1.360 (5)	C8—C9	1.399 (3)
C2—C3	1.382 (4)	C9—O1	1.334 (3)
C2—H2	0.9300	C9—C10	1.396 (3)
C3—C4	1.381 (4)	C10—C11	1.373 (4)
C3—H3	0.9300	C10—Br1	1.878 (3)
C4—C5	1.387 (4)	C11—C12	1.379 (4)
C4—N1	1.418 (3)	C11—H11	0.9300
C5—C6	1.386 (4)	C12—C13	1.370 (3)
C5—H5	0.9300	C12—Cl1	1.741 (3)
C6—H6	0.9300	C13—H13	0.9300
C7—N1	1.270 (3)	O1—H1	0.8200
C7—C8	1.460 (3)		
			
F1—C1—C6	118.6 (3)	C13—C8—C9	120.0 (2)
F1—C1—C2	118.5 (3)	C13—C8—C7	119.4 (2)
C6—C1—C2	122.8 (2)	C9—C8—C7	120.5 (2)
C1—C2—C3	118.3 (3)	O1—C9—C10	119.7 (2)
C1—C2—H2	120.9	O1—C9—C8	122.2 (2)
C3—C2—H2	120.9	C10—C9—C8	118.1 (2)
C4—C3—C2	120.9 (3)	C11—C10—C9	122.0 (2)
C4—C3—H3	119.6	C11—C10—Br1	119.22 (18)
C2—C3—H3	119.6	C9—C10—Br1	118.82 (19)
C3—C4—C5	119.2 (2)	C10—C11—C12	118.6 (2)
C3—C4—N1	116.1 (2)	C10—C11—H11	120.7
C5—C4—N1	124.7 (2)	C12—C11—H11	120.7
C6—C5—C4	120.0 (3)	C13—C12—C11	121.5 (2)
C6—C5—H5	120.0	C13—C12—Cl1	120.1 (2)
C4—C5—H5	120.0	C11—C12—Cl1	118.38 (19)
C1—C6—C5	118.8 (3)	C12—C13—C8	119.8 (2)
C1—C6—H6	120.6	C12—C13—H13	120.1
C5—C6—H6	120.6	C8—C13—H13	120.1
N1—C7—C8	121.3 (2)	C7—N1—C4	122.7 (2)
N1—C7—H7	119.3	C9—O1—H1	109.5
C8—C7—H7	119.3		
			
F1—C1—C2—C3	178.5 (3)	O1—C9—C10—C11	179.2 (3)
C6—C1—C2—C3	−1.0 (5)	C8—C9—C10—C11	−1.1 (4)
C1—C2—C3—C4	0.1 (5)	O1—C9—C10—Br1	−1.2 (3)
C2—C3—C4—C5	0.8 (4)	C8—C9—C10—Br1	178.50 (19)
C2—C3—C4—N1	−177.8 (3)	C9—C10—C11—C12	0.8 (4)
C3—C4—C5—C6	−0.9 (4)	Br1—C10—C11—C12	−178.8 (2)
N1—C4—C5—C6	177.6 (2)	C10—C11—C12—C13	0.3 (4)
F1—C1—C6—C5	−178.6 (3)	C10—C11—C12—Cl1	−179.7 (2)
C2—C1—C6—C5	1.0 (5)	C11—C12—C13—C8	−0.9 (4)
C4—C5—C6—C1	0.0 (4)	Cl1—C12—C13—C8	179.0 (2)
N1—C7—C8—C13	177.0 (3)	C9—C8—C13—C12	0.6 (4)
N1—C7—C8—C9	−2.6 (4)	C7—C8—C13—C12	−179.0 (2)
C13—C8—C9—O1	−179.9 (2)	C8—C7—N1—C4	−178.1 (2)
C7—C8—C9—O1	−0.3 (4)	C3—C4—N1—C7	−170.8 (3)
C13—C8—C9—C10	0.4 (4)	C5—C4—N1—C7	10.7 (4)
C7—C8—C9—C10	180.0 (2)		

### Hydrogen-bond geometry (Å, °)

**Table:**

*D*—H···*A*	*D*—H	H···*A*	*D*···*A*	*D*—H···*A*
C11—H11···F1^i^	0.93	2.45	3.349 (4)	162
O1—H1···N1	0.82	1.86	2.577 (3)	146

Symmetry codes: (i) *x*+1, *y*−1, *z*+1.
